# Post-traumatic growth of people who have experienced earthquakes: Qualitative research systematic literature review

**DOI:** 10.3389/fpsyt.2022.1070681

**Published:** 2023-03-02

**Authors:** Hyun-Ok Jung, Seung-Woo Han

**Affiliations:** ^1^College of Nursing, The Research Institute of Nursing Science, Daegu Catholic University, Daegu, South Korea; ^2^Department of Nursing, Kwangju Women's University, Gwangsan-gu, Gwangju, South Korea

**Keywords:** systematic review, post-traumatic growth, qualitative research, earthquakes, disaster

## Abstract

**Introduction:**

Earthquakes can have a variety of physical, emotional, and social effects on the people who experience them. Post-traumatic Growth (PTG) results from people attempting to reconstruct their lives after experiencing a traumatic event. We intend to inform the local community of the importance of disaster psychology by identifying and analyzing the literature on post-traumatic growth experiences of subjects who experienced earthquakes.

**Methods:**

This study applied a systematic review of qualitative research published from January 1, 2012 to January 31, 2021 to understand PTG in people who have experienced earthquakes. The search expressions “Post-traumatic Growth”, “Earthquake”, “Qualitative” were applied to CINAHL, EMBASE, PubMed, PsycInfo, KISS, RISS, and NDSL databases. Initially, 720 papers were found; after removal of duplicates, 318 remained. After a review of titles and abstracts, 186 papers that did not meet the selection criteria of this study were removed. After a further examination of the remaining 132 papers, the researchers removed 65 papers that did not match the research topic. Lastly, of the remaining 67 papers, detailed review eliminated quantitative papers that did not match this study (25), articles that were not original (19), articles in which results were not PTG (8), articles that were not related to this study (3), articles that were not written in English (2), or articles that had mixed topics (2). Eight papers remained.

**Results:**

The results of this study show that the PTG in people who have experienced earthquakes can be classified into three categories: “Change in self-perception”, “Change of interpersonal relationships”, and “Spiritual change”. They can be further classified into eight subcategories: “Reviewing one's existence”, “Acceptance”, “Discovering strengths by working through adversity”, “Gratitude for life”, “Changes in personal relations”, “Changes in social relations”, “Accepting the existence of God”, and “A breakthrough to overcome difficulties”.

**Discussion:**

These results can be used as basic data for a positive psychological understanding for those who have experienced earthquake trauma.

## Introduction

Earthquakes are unpredictable and uncontrollable as they occur suddenly, often without warning (1). In the past year, 103 earthquakes have occurred in the New Caledonia Noumea region of the South Pacific, including one with a magnitude of 7.9. Furthermore, 77 earthquakes with a magnitude of 2.0 or higher have occurred in South Korea (2). Earthquakes affect humans in a variety of physical, emotional and social ways. Physical effects include various degrees of injury. Emotional effects include anxiety, fear, anger, and depression. Social effects include loss of infrastructure, destruction of communities and workspaces, and damage to the natural environment (1). Through these effects, earthquakes can have a transformative effect on a person's life (1, 3). Natural disasters such as earthquakes are traumatic events that humans did not intend, which inflicts pain on individuals' lives, but in the field of changed life, humans experience another growth and recovery (4).

Post-traumatic growth has been actively studied in various population groups. In a study on post-traumatic growth of college students who experienced an earthquake, depression was found to be a factor influencing post-traumatic growth (5). A study on childhood who experienced natural disasters showed that the level of post-traumatic stress following the disaster affects post-traumatic growth (6). This suggests that emotional states or pain, such as depression or level of post-traumatic stress, are catalytic factors for overcoming the negative psychological consequences of traumatic events. Various traumatic experiences can also influence mental disorders in childhood. In previous study (7), traumatic experiences in childhood cause various mental health problems. Approximately 20.7% of them had psychotic-like experiences as adults, and 17.5% had frequent delusional experiences. Considering the results of previous studies, it is believed that traumatic experiences in childhood can have a negative impact on mental health even in adulthood, and various therapeutic interventions should be accompanied. Accordingly, personal protective factors (resilience, depression) and social protective factors (Household income and educational level) have been reported as factors that can positively mediate responses to traumatic events (5, 6, 8, 9).

Calhoun and Tedeschi, who proposed the post-traumatic growth model (10), argued that in order to experience post-traumatic growth, the psychological pain induced by the traumatic event and the collapse of individual core beliefs were required. In other words, post-traumatic growth is closely related to negative psychological conditions such as post-traumatic stress disorder, and the representative symptoms of post-traumatic symptoms are intrusion, avoidance, and hyperarousal (11). As a result of studying the post-traumatic growth of terrorist survivors who experienced PTSD (12), emotional numbing of survivors was found to be related to post-traumatic growth after about 6–12 months, so it is necessary to study various psychological symptoms induced by PTSD. However, it was mentioned that humans do not always accept pain negatively, but rather try to resolve traumatic experiences more positively and goal-focused on the basis of resilience (10). The importance of positive coping strategies should continue to pay attention.

Post-traumatic Growth (PTG) is an improvement in mental health that occurs while a person develops a better understanding of the meaning of traumatic events, and starts to gain hope for life (6, 13). Post-traumatic growth tool development research includes personal strength, new possibilities, relating to others, appreciation of life, and spiritual change (14, 15).

The repetitive mental revisiting of the traumatic event by the traumatized person changes cognitive processes, and unpleasant feelings experienced by trauma act as motivations to move forward from the event (3), and therefore the traumatized persons shows a positive attitude toward understanding themselves, others, and life in general (16). Therefore, the traumatized person gains confidence that he or she is capable and strong (1, 17), and starts to make efforts to know the importance of not returning to the pre-traumatic period. They also try to live a better life by realizing the meaning of life, by finding good behavior to achieve the life they want to pursue, and by inducing positive changes such as escaping from bad behavior (3). Therefore, PTG is an active and positive process that restructures individual lives to pursue better independent lives (3, 18). If common growth experiences of traumatized people can be understood, community health professionals can help traumatized patients escape from pain and return to their pre-traumatic lives.

In this study, a systematic review of qualitative research was conducted to understand the PTG experience of subjects who had experienced earthquakes. A phenomenological research method is applied to derive meaningful subjective interpretations to understand the positive psychological changes of people traumatized by earthquakes. Furthermore, this study intends to lead a quality life by finding the meaning of life through post-traumatic growth and forming a sense of purpose to rebuild a new life.

## Materials and methods

### Study design

This study performs a systematic review to search for previous papers and evaluate their quality to ensure that they represent the PTG experience of subjects who have experienced earthquakes.

### Research protocol

The purpose of this study is to systematically review the literature of qualitative studies that have studied the post-traumatic growth experiences of subjects who have experienced earthquakes. Based on the qualitative evaluation protocol of the qualitative research, the CASP (Critical Appraisal Skills Program) qualitative research checklist (19) was used. This CASP qualitative research checklist extracts data based on the following 10 systematic protocols. (1) Is there a clear description of the research goal? (2) Is the methodology for qualitative research appropriate? (3) Study design (4) Recruitment strategy (5) Appropriate data collection method (6) Relationship between researcher and subject (7) Ethical issue (8) Analysis method (9) Is there a clear description of the results? (10) research value.

### Literature search and literature selection

In this study, to search for qualitative literature on the experiences of post-traumatic growth of subjects who experienced earthquakes, the literature was searched through foreign databases CINAHL, EMBASE, PubMed, and PsycInfo and domestic databases KISS, RISS and NDSL. Overseas databases checked the terms of MeSH and extracted all of the terms “Post-traumatic Growth”, “Earthquake”, and “Qualitative Research” as intervention methods.

According to the characteristics of each database, MeSH terms and text words were used for the search formula, and methods to increase the specificity in addition to the sensitivity of the search were used by applying the Boolean operators AND/OR and truncation search. The domestic database search was based on the search strategy used for overseas searches, but considering the lack of a MeSH search function, the search was conducted according to the characteristics of each database. As the keywords for the search, concepts such as post-traumatic growth, earthquake, and qualitative research were searched and extracted. The literature selection criteria were (1) qualitative research on post-traumatic growth of earthquake-experienced individuals, (2) papers published in the last 10 years from January 1, 2012 to January 31, 2021, (3) In case of overlap between academic research paper and degree thesis, academic research paper was selected and (4) academic research paper composed in English and Korean was included. The exclusion criteria were (1) papers using words similar to post-traumatic growth (e.g., psychological adaptation and resilience), (2) papers published before and after between January 1 2012 and January 31, 2021, (3) papers related to natural disasters other than earthquakes (e.g., floods, forest fires, etc.), (4) papers not published in English or Korean.

### Data collection

**Figure 1:**
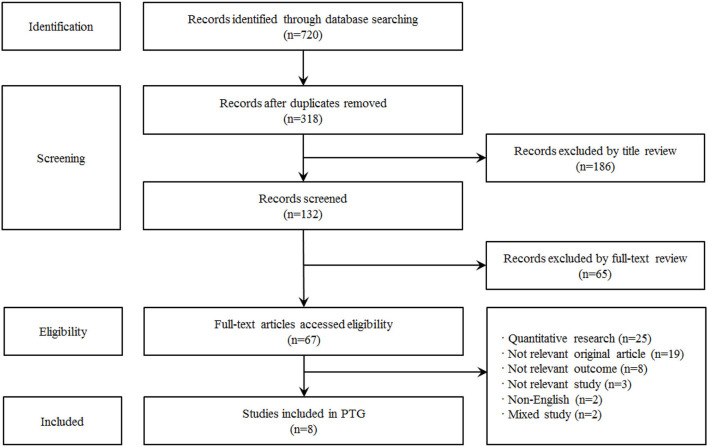
Flow chart of the sample selection process.

In this study, the literature was selected according to the selection and exclusion criteria, and the selection process was described in the following stages: Identification → Screening → Eligibility → Included. A total of 720 documents were searched in the database, and 318 articles were derived after removing 402 documents that were duplicated. Two researchers reviewed the title and abstract of a total of 318 articles, and 132 articles were first selected, excluding 186 articles that did not meet the selection criteria of this study. Of the 132 papers that were reviewed according to the same criteria and process, mainly the original text, 65 papers that did not match the research topic were excluded through three meetings, and 67 papers were secondarily selected. Finally, cross-analysis was performed twice on 67 documents that researchers secured the suitability of the study. The final 8 papers were selected except for the quantitative papers (25 papers), non-original articles (19 papers), post-traumatic growth papers (8 papers), papers (3 papers), non-English papers (2 papers), and mixed papers (2 papers) that did not correspond to this study. The searched literature was independently performed by two researchers, and the final paper was selected through discussion in case of disagreement (Figure 1).

### Ethical consideration

This study was approved by the K University Institutional Review Board (IRB No: 1041459-202103-HR-004-01) as it complied with research ethics in the use of literature data.

## Results

### Assessment of research quality

This research generally used the Critical Appraisal Skills Program (CASP) to evaluate the quality of qualitative research; CASP is an effective means of improving the understanding of individual studies (20). The final selection of papers was evaluated using CASP, which determined that all met 23 to 26 out of 28 items, and therefore were appropriate for use in this study. Specifically, two papers did not conform to “Qualitative methodology for question?”, two papers did not conform to “Discussed saturation of data?”, and five were inconsistent in the item “Critically examined the role, potential bias and influence during data collection?” These results suggest that the biases that are caused during data collection in future qualitative studies should be closely examined. Two papers did not meet the items “Sufficient details of how the research was explained to participants” and two did not meet “Approval sought from an ethics committee?” This result suggests that compliance with research ethics should be considered as being important in qualitative research. One paper did not meet the “In-depth description of the analysis process?” item, and one did not meet the “Sufficient data presented to support the findings?” Six papers that did not meet the item of “Contradictory data taken into account?”, and CASP evaluation showed that this item was the most frequently violated. This observation implies that bias in research should be minimized by specifically stating and reviewing contradictory data in qualitative research. Finally, two papers did not meet “Discussed evidence for and against research's arguments?” and “Discussed contribution study makes to existing knowledge?” (Table 1). We then checked the systematic literature review based on the PRISMA checklist (21).

**Table 1:** Quality assessment (CASP, Critical Appraisal Skills Programme).

**CASP Checklist**	**[1]**	**[2]**	**[3]**	**[4]**	**[5]**	**[6]**	**[7]**	**[8]**
1. Clear statement of the aims of the research?	Y	Y	Y	Y	Y	Y	Y	Y
2. Qualitative methodology for question?	Y	Y	Y	N	Y	N	Y	Y
3. Research design appropriate to address the aims of the research?	Y	Y	Y	Y	Y	Y	Y	Y
4a. Explained how participants were selected?	Y	Y	Y	Y	Y	Y	Y	Y
4b. Explained why participants selected were appropriated?	Y	Y	Y	Y	Y	Y	Y	Y
4c. Discussed recruitment (e.g., why some chose not to take part?)	Y	Y	Y	Y	Y	Y	Y	Y
5a. Justified setting for data collection? (e.g., focus group, interview?)	Y	Y	Y	Y	Y	Y	Y	Y
5b. Clear how data were collected (e.g., focus group, interview?)	Y	Y	Y	Y	Y	Y	Y	Y
5c. Justified the methods chosen?	Y	Y	Y	Y	Y	Y	Y	Y
5d. Data collection methods explicit?	Y	Y	Y	Y	Y	Y	Y	Y
5e. From of data clear (e.g., tape recordings, video material, notes?)	Y	Y	Y	Y	Y	Y	Y	Y
5f. Discussed saturation of data?	N	N	Y	Y	Y	Y	Y	Y
6a. Critically examined role, potential bias and influence during data collection?	N	Y	N	N	N	Y	N	Y
7a. Sufficient details of how research was explained to participants	Y	Y	Y	Y	N	Y	N	N
7b. Issues around informed consent or confidentiality described?	Y	Y	Y	Y	N	Y	Y	Y
7c. Approval sought from an ethics committee?	Y	Y	N	N	Y	Y	Y	Y
8a. In-depth description of the analysis process?	N	Y	Y	Y	Y	Y	Y	Y
8b. Clear how the categories/themes were derived from the data?	Y	Y	Y	Y	Y	Y	Y	Y
8c. Explained how the data presented were selected from sample?	Y	Y	Y	Y	Y	Y	Y	Y
8d. Sufficient data presented to support the findings?	Y	Y	Y	Y	Y	Y	N	Y
8e. Contradictory data taken into account?	N	N	Y	Y	N	N	N	N
9a. Finding explicit?	Y	Y	Y	Y	Y	Y	Y	Y
9b. Discussed evidence for and against research's arguments?	Y	Y	Y	Y	Y	Y	N	N
9c. Discussed credibility of findings (e.g., triangulation, >1 analyst)?	Y	Y	Y	Y	Y	Y	Y	Y
9d. Findings discussed in relation to research question?	Y	N	Y	Y	Y	N	Y	Y
10a. Discussed contribution study makes to existing knowledge?	Y	Y	Y	Y	Y	Y	Y	Y
10b. Identified new areas where research is necessary?	Y	Y	Y	Y	Y	Y	Y	Y
10c. Discussed applicability of findings?	Y	Y	Y	Y	Y	Y	Y	Y
Total (Percentage of Yes)	24/28	25/28	26/28	25/28	24/28	25/28	23/28	25/28

### Characteristics of literature subject to systematic literature review

The eight papers that were ultimately selected in this study were all published from 2015 to 2020, and most focused on subjects in the South Pacific and Southeast Asia, where earthquakes occur frequently. Interview duration ranged from 30 to 240 min (or no duration was provided), and the action was described as a revisit to ask a question or an additional question. The number of subjects studied ranged from four to 23, and included both males and females, although one paper did not specifically mention the gender(s) of the participants. The ages of the subjects ranged from the teens to the sixties. Occupations were middle and high school students, college students, nurses, and psychiatric specialists. Data collection used semi-structured interviews in five cases, and in-depth interviews in three cases (Table 2).

**Table 2:** Demographic characteristic.

**No**	**Author**	**Country**	**Interview duration (Min)**	**Sample size (n)**	**Mean age (year)**	**Job**	**Date collection method**
1	Johal et al. (22)	New Zealand	39–70	1	49–64	Nurses	Semi-structured interview
				Male: 1			
				Female: 10			
2	Jaimes et al. (23)	Haiti	45–150	22	28–64	Local	Semi-structured interview
				Male: 6		clinicians	
				Female: 16			
3	Hwang and Ra (24)	Nepal	60	15	25–38 years	University,	Semi-structured interview
				Male: 8		Graduate students	
				Female: 7			
4	Salawali et al. (3)	Indonesia	30–60 min	16	14–18	Adolescent	In-depth interview
				Male: 3			
				Female: 13			
5	Ren et al. (25)	China	45–90	23	25–62	Psychiatric specialists	Semi-structured interview
**6**	Iguchi et al. (26)	Japan	105–240	7	20s−50s	Public health	In-depth interview
				Male: 1		nurse	
				Female: 6			
**7**	Pratt et al. (1)	New Zealand	None	4	10s	Senior	Semi-structured interview
				Male: 1		students	
				Female: 3			
**8**	Wijoyo et al. (27)	Indonesia	45–60	14	21–56	Nurses	In-depth interview
				Male: 7			
				Female: 7			

### Post-traumatic growth experience of earthquake-experienced people

The papers finally selected in this study were assigned to three categories and eight subcategories encompassing the PTG experiences of subjects who had experienced an earthquake (Table 3).

**Table 3:** Categories and subcategories of PTG response.

**Category**	**Subcategory**	**Attribute**
Change in self-perspective	Reviewing one's existence	Change in attitude toward life (3)
		Limit of existence (5)
		Realizing the meaning of life (4)
		Clarification of the value of life (1)
	Acceptance	Uncertainty of life (5)
		Acceptance of feelings of pain and loss (6)
	Discovering strengths by working through adversity	New challenge (1,5,2)
		Pride (1)
		Flexibility (7)
		Occupational consciousness (5)
	Gratitude for life	Lucky to be alive (1)
		An unforgettable experience in life (1)
Change of interpersonal relationship	Changes in personal relations	Strong family solidarity unlike before (6)
		Reduction of prejudice against others (3)
		Understand the pain of others (2)
		Increased solidarity with colleagues (6)
	Changes in social relations	Increased community solidarity (6)
		Friendliness with residents (6)
Spiritual change	Accepting the existence of God	Disaster is the process of approaching God (8)
		Expanding the Perspective of Religion
		and Spiritual Being (7)
	A breakthrough to overcome difficulties	The power of religion (5)
		A resting place to lean on (3)

### Change in self-perception

The first category of responses identified in this study is a change in self-perception. By reflecting on the world in which they live and by changing their values and philosophy on life, the traumatized people reflected on the meaning of life and became aware of their existence. This change meant the subjects who had experienced earthquakes found their inner strength and could accept their current life and overcame the adversity that they had experienced.

#### Reviewing one's existence

Some subjects who experienced PTG after an earthquake underwent a change in attitude toward life. Before the event, they thought they were just ordinary people, but afterward, they realized their own importance. They also realized the limitations of their existence in that they could not do anything in the face of an earthquake, and experienced a change in life priorities.

“*Many things that had previously seemed to be important in the past are no longer important. After experiencing an earthquake, I felt that I should value myself more and I realized that I was precious to others. I've lived for others, but from now on I want to live a more valuable life for myself because you only get one chance at life*.” (22, 24)

“*My view of the world has suddenly changed. The world is not as gentle as I thought, the earth can move under my feet, and buildings can collapse around me at any point. The earthquake made me realize so many things; that human beings are weak, small, and not omnipotent. I want to be a person who has a purpose in life because I live to die and I am always ready to die...”* (3, 25)

#### Acceptance

Most of the subjects who experienced the earthquake realized that the place where they lived was not safe: that it could take away someone's life in an instance, and they learned the importance of accepting this fact as a part of life.

“*I try to think of pain as a normal part of life because life is not about letting go of the pain, but about carrying it with you. So life has no answer. It would be more comfortable if the answer was given to you. I couldn't express my sad feelings after experiencing the earthquake. I can't erase the thought that the residents who had been with me disappeared one by one and lost what they had built up. But I thought I shouldn't feel pain because my family was safe*.” (25, 26)

#### Discovering strengths by working through adversity

Some subjects who underwent PTG after experiencing an earthquake said that the experience of an earthquake did not emphasize human weakness. They felt that humans were stronger than they thought because they did not die even though they had experienced a terrifying environment. They had learned that their strengths were not simply limited to physical strength, but also include the inherent tendency, intrinsic flexibility, confidence, and occupational consciousness that all humans possess.

“*After experiencing the earthquake, I became more positive because seeing the collapsed city rebuild itself and thinking about what was going to happen now and, in the future, made things less confusing and more hopeful. I saw the difficult things, but I also saw the beautiful things. I have learned a lot. Living by overcoming this situation had made me face new challenges, and now I believe I can do anything.”* (1, 22, 23)

“*Looking back on whether I really responded well to others after the earthquake made me realize a lot and helped me grow my professional expertise as well as my own personal growth. Also, after the earthquake, I had learned how to deal with uncontrollable situations during work. I was impressed to see people recover one by one. By knowing that patients can recover from their pain, we have learned that we should not overlook anything for them”* (25, 26)

#### Gratitude for life

Some subjects who experienced PTG after the earthquake showed a positive change compared to life before the earthquake.

“*I wasn't hurt or killed and the house wasn't badly damaged. Although I haven't been hurt, I think it's a very amazing experience to be with other people who have been hurt. The earthquake was a pretty good experience for me, and I think it was an opportunity to remind me of my existential appreciation for life. Through this experience, I want to look back on myself and live my life with gratitude for everything*.” (22, 23)

### Change of interpersonal relationship

Some subjects who went through PTG after experiencing an earthquake changed positively in realizing that people should develop mutual relations with others, and help and support each other. These subjects showed more active behaviors such as taking care of family, friends, and colleagues, and showed empathy with people in need.

#### Changes in personal relations

Some subjects who underwent PTG after the earthquake experienced a change in their interpersonal relationships completely different from before the earthquake.

“*I have a reduced prejudice against others and my neighbors. Before, I had a prejudice against people from other regions or people from other religions, and after experiencing the earthquake, I tried to see the positive aspects of those people. I realized that the pain of others was my pain because the whole country suffered. In the end, shared experiences with people who experienced earthquakes has given me an opportunity to experience hope, solidarity, learning and growth as well as pain.”* (23, 24)

“*After the earthquake, I realized many things. When I go to work I feel like I'm the only one working hard, and I wondered why my colleagues wouldn't work hard. But I noticed that they also worked hard and did their best. We survived and we are still working hard. Through this, we could feel a different sense of fellowship than before. In the past, I was just resting at home doing nothing, but now I help my parents and cook for them. I realized that what really matters is my relationship with my family, friends and colleagues.”* (3, 22, 26)

#### Changes in social relations

Some subjects who experienced PTG after an earthquake then viewed social relations completely differently compared to before the earthquake. Community solidarity with other communities increased and people felt friendliness with residents. In addition, the earthquake experience allowed subjects to feel the culture of helping each other unlike before, so they experienced many changes in the form of social networking.

“*After the earthquake, I could feel that my intimacy with my neighbors increased and the community was strengthening. The earthquake brought us together and allowed us to feel the atmosphere of harmony and cooperation. Also, I was able to cooperate with people from other professions, and I was able to help people who are more in need than I am*.” (24, 26)

“*I give up my things to others, and I am not only thinking about myself or my family, but also other people. After the earthquake, I contacted people with who I had a distant relationship with and encouraged them to participate in aid agencies. Also, I made frequent contact with nurses at local hospitals and cooperated with them when they were having difficulties. We were able to talk directly to local group staff who had no involvement before the earthquake. So am now more capable of helping people who are in a more difficult situation than me compared to before the earthquake*.” (3, 26)

### Spiritual change

The third essential theme of the final selected papers in this study is spiritual change. Some people who experienced the earthquake have broadened their religious and spiritual perspectives and felt that the experience of the earthquake was a step toward God. After all, it was an experience that reminded me once again that being alive in an earthquake was the same as if God was alive.

#### Accepting the existence of god

For some people, the experience of the earthquake increased their faith in religion and God, because they believed that they survived the earthquake because God had helped them. They also said that the experience of an earthquake was a part of the process of approaching God. In the end, it was an opportunity to experience the ability of humans to live with God, and that religion is not a metaphysical point of view, but an existential point of view.

“*After a disaster, my chance to live is a gift from God. After seeing what the Lord is doing for us, I started praising God. After experiencing the earthquake, I think the disaster has become a channel to connect with God. In doing so, I think the relationship between me and God has been further strengthened.”* (1, 27)

#### A breakthrough to overcome difficulties

Among the subjects who experienced an earthquake, those who experienced PTG experienced the power of religion differently. Those with religion were more flexible than those without religion in their coping attitude to overcome difficulties and experienced the difficult moments (such as facing death) through God.

“*I feel the power of religion, and I can overcome the trauma through religion. I have religion on the basis of my life as a whole and I can overcome my difficulties through faith.” My environment, based on the influence of my parents and my religious life, helped me to overcome and overcome the earthquake even after the earthquake. If you have faith, you will find Paradise as a reward for your difficulties*.” (24, 25)

## Discussion

This study systematically applied a phenomenological research method to understand PTG in subjects who had experienced earthquakes and discussed the categories and subcategories derived from this study.

“Reviewing one's existence” and “Acceptance”, which are subcategories of “Change in self-perception”, reveal the experience of subjects having a change in their view of life and philosophy of life after the earthquake. Also, by discovering strengths while undergoing adversity, and finding gratitude for life, they sublimated their painful traumatic experience into strengths, so the experience of the earthquake was not solely a bad one, but an opportunity to discover that they were grateful just to be alive.

Post-traumatic growth is the result of individuals' cognitive and emotional efforts to treat and give meaning to natural disasters as events in their difficult lives. At this time, fear leads to finding the meaning of life in the event of trauma, and the rumination of questions about life's doubts is later converted to rumination of questions about the meaning of life. Stronger self-confidence and new beliefs are reconstructed (4).

In previous studies, it was said that human experience of a terrible traumatic event creates new wisdom and experiences post-traumatic growth through the rumination process of thinking about the impact and meaning on one's own existence and life (23). In the end, by reflecting on one's own existence, it recognizes the optimal direction of life and activates aspiration and hope. This is not a life cycle in which mental suffering through trauma simply falls into a bad abyss. It is to positively accept information related to the new trauma in the meaning and purpose of being in one's life (27).

In this study, subjects who experienced earthquakes also accepted earthquakes as an unavoidable fate, so just being alive was a great luck and gratitude for their lives, and they recognized earthquakes as good experiences in life. This study result is consistent with the result that the factor of gratitude (Factor V) was statistically significant even in the study of the author who developed the PTG tool (28).

In particular, “An appreciation for the value of my own life” was 0.85 among the life's gratitude factors, and it was the item most related to post-traumatic growth. After all, the experience of earthquakes can be regarded as a starting point that makes people think about their existential gratitude once again.

We can think about it once again. What could be the cause of these positive emotions? This is because some people experience post-traumatic stress, not post-traumatic growth, after experiencing trauma. In previous study (29), it was found that in people who survived traumatic events, internal emotions such as guilt and self-deprecation cause post-traumatic stress on the contrary. In this study, subjects who experienced an earthquake also experienced internal suppression in situations in which they were unable to express their shock, fear, and sadness at the death of their colleague. However, they were able to experience post-traumatic growth because human strength existed even in pain. In this study, strength was expressed as flexibility and confidence. It is consistent with this study in that previous study (30) also mentioned that internal strength factors such as strength and flexibility are triggers that can promote post-traumatic growth and overcome pain. In addition, in the study (28) of the author who developed the post-traumatic growth tool, the “Knowing I can handle difficulties” item in the personal strength factor (Factor III) was 0.79, which is the same as the result that it is related to post-traumatic growth the most.

In traumatic events, PTG and pain coexist. Dealing with the pain and threats to life's worth of traumatic events requires a lot of time and cognitive effort. Through the repetitive process of understanding and trying to understand the traumatic event experience, human suffering is reduced by experiencing positive psychological changes and meaning to life. In addition, the value and meaning of a new life are integrated, leading to a higher standard of life (31).

In the second essential theme of this study, 'changes in interpersonal relationships', earthquake victims experienced various changes in personal and social relationships. They demonstrated a reduction in prejudice against others who have different values, and that their value of family, friends, and colleagues increased. In addition, as community solidarity with other communities increased, the experience of cooperative social consciousness changed.

Some subjects who experienced the earthquake realized that the pain experienced during the earthquake was shared by all who experience it. This shared experience between people who had survived the earthquakes allowed people to recognize that they never lived alone, helping them develop a sense of solidarity with others, and gratitude toward family, friends, and colleagues. As a result, in serious situations such as natural disasters, others support trauma experienced people with a sincere heart. Trauma survivors realize or experience the meaning of life through satisfactory relationships, and form close relationships with others by expanding and deepening interpersonal relationships. In particular, this is because, as traumatized people get social support from meaningful relationships, their philosophy of life has changed, reconstructing their meaning system, and more effectively participating in the emotional and cognitive processes of post-traumatic growth (4). In the previous study (32), it is consistent with the results of the study that talking and sharing trauma experiences with others increases intimacy with others and improves understanding and empathy for others suffering. In addition, in the study (28) of the author who developed the post-traumatic growth tool, the “A sense of closeness with others” item in the Relating to Others factor (Factor I) was 0.81, which is the same as the result that it is related to post-traumatic growth the most. Through the earthquake experience, the subjects who experienced pain recognized the importance of human relationships that were different from before. In addition, the formation of social networks, such as cohesion different from those before the earthquake experience, allows earthquake survivors to reconstruct the meaning of the experience and recognize the potential benefits of this experience. In doing so, the experience of events is sublimated positively, improving relationships with others, and creating new life possibilities to experience positive psychological changes (33). Therefore, through the changed interpersonal relationships, traumatized people discovered a new form of meaning for life after trauma, and formed a sense of purpose to rebuild a new life (31).

In the spiritual change, which is the last essential theme in this study, earthquake experienced a change in recognizing the existence of God and reflecting on the meaning of religion unlike before. After experiencing an earthquake, these people used religion as a method to overcome difficulties and tried to rely on God to solve problems that they could not overcome. Those who had survived earthquakes cast their survival as evidence of a link between themselves and God, and stated that they had become closer to God by surviving. In doing so, they became more convinced of the existence of God and also experienced a change of spiritual emotions. In previous studies (28, 34), spirituality is the factor that has the greatest influence on post-traumatic growth, and through loss, we question our spiritual beliefs. Those who had experienced a traumatic event developed the belief that everything that happened around them was God's spirituality. They had accepted the traumatic event, and this acceptance had led to PTG. This observation is consistent with the results of this study in that by accepting the existence of God and one's relationship with God, one's religious beliefs can become more firmly established (28). Because human beings are complex beings with interrelated physical, psychological, social, and spiritual capacities, a holistic understanding of traumatic events must incorporate religion and spirituality. Traumatic events not only endanger the physical, psychological and social wellbeing of a person, but also have a powerful impact on the spiritual wellbeing (35–37).

In previous studies (36, 37), it is consistent with the research results that traumatized people tried to cope after a crisis through spirituality, and through this, they supported God and positively overcome difficulties.

Religion and spirituality affect not only people's perception of life events and their initial evaluation of traumatic events, but also their chosen coping methods, coping functions, and coping outcomes (35). Positive religious and spiritual coping methods are secure connections with God, self, and others, including: (1) finding meaning, (2) gaining dominance and control, (3) comforting and increasing intimacy with God, (4) increasing intimacy with others and intimacy with God, (5) achieving life change.

On the other hand, negative religious spiritual coping methods attempt to resolve the five-positive religious spiritual coping functions related to conflicts with God, self, and others, but rather worsen post-traumatic pain (38, 39). Earthquake survivors use traumatic events as evidence of God's perfect and mystical will, “for good reason,” or as an opportunity for change in their spiritual growth. In addition, it is interpreted as a spiritual challenge or a test of God's devotion and seeks a partnership to solve problems in cooperation with God by utilizing positive religious and spiritual coping methods. In doing so, gaining dominance and control over disasters, finding a new meaning in life, and forming a sense of purpose for a changed life (35). This study result is consistent with the result that the factor of Spiritual Change (Factor I) was statistically significant even in the study of the author who developed the PTG tool (28). In particular, “A better understanding of spiritual matters” was 0.84 among the Spiritual Change factors, and it was the item most related to post-traumatic growth.

As a result of this study, changes in self-perspective, changes in interpersonal relationships, and spiritual changes are all included in the five areas (new possibilities and personal strength, relating to others, spiritual change, appreciation of life) of Tedeschi and Calhoun's PTG tool (28). When humans experience disasters such as earthquakes, they understand the difficult situation and changed reality they face, and find their strengths in the belief that they are strong.

And as they establish new relationships with others, they realize that the most important thing in life is themselves, and they regain the meaning of a new life given to them after the trauma. Gratitude for a new life every day, through stronger religious beliefs, makes spiritual changes and leads to a positive life.

Finally, in a situation where research on post-traumatic growth is being actively conducted, there was a verification of the post-traumatic growth tool using a variety of population groups as samples. However, it was found that the factor structure differed depending on the country that justified the post-traumatic growth tool. Also, some items did not significantly measure post-traumatic cultural change (40). Therefore, in order to overcome the limitation that cannot objectively express post-traumatic growth in qualitative research, demographic characteristics or cultural aspects of the country should be considered. Based on the research results from qualitative research, it is considered that it is necessary to continuously supplement the weaknesses of the tool through more practical and multifaceted analysis.

## Limitation and future direction

This study does however have some limitations. This study presented papers that studied subjects who experienced the special situation of earthquakes among natural disasters. The results of this study have limitations in covering the post-traumatic growth of subjects who have experienced all-natural disasters. Also, because the research topic was very special, the number of published studies was very limited, and most of the studies were published in a limited country where earthquakes occur frequently in the region. Nevertheless, this study is considered to be very valuable in approaching the actual field phenomena of the post-traumatic growth of earthquake-experienced people by examining the qualitative studies on post-traumatic growth. In the special situation of earthquakes among natural disasters, post-traumatic growth experience analysis can provide basic data for the development of psychological intervention programs in regions where earthquakes frequently occur. So far, systematic literature review has been focused on quantitative research, but qualitative research is very lacking. In particular, in quantitative research, guidelines for evaluating the quality of various documents and data extraction for systematic literature review are presented, but in qualitative research, they are very limited. Therefore, it will be necessary to develop various evaluation tools for qualitative research in future research.

## Conclusion

This study systematically reviewed published papers that explored PTG in subjects who experienced earthquakes. It then categorized the different PTG phenomena and their individual significance. This study identified that PTG can involve changes in self-perception, interpersonal relationships, and religious beliefs. The subjects of the research papers changed their views on the value of life by reflecting on their existence rather than by struggling to escape the pain they felt. They also experienced increased appreciation for life as they embraced the experience and overcame their adversity. In addition, they placed more value on the importance of human relationships and felt a sense of solidarity. Finally, some subjects experienced a spiritual change where they realized the meaning of religion and affirmed their belief in the existence of God.

People who experienced the earthquake modified their values of life through traumatic events. Finding the meaning of a new life and examining one's own life promoted growth in areas such as personal strength, relationships with others, gratitude for life, and spirituality. Community health professionals should recognize that post-traumatic growth is a cognitive and emotional result of seeking a new life, and provide opportunities for earthquake victims to discover new forms of meaning in their lives sequentially. In addition, it should be helped to maintain a higher level of life satisfaction through addition or modification of the sense of purpose.

## Data availability statement

The original contributions presented in the study are included in the article/supplementary material, further inquiries can be directed to the corresponding author.

## Ethics statement

This study was approved by the K University Institutional Review Board (IRB No: 1041459-202103-HR-004-01) as it complied with research ethics in the use of literature data.

## Author contributions

H-OJ: conceptualization and investigation. S-WH: methodology and data curation. H-OJ and S-WH: writing-original draft preparation and writing-review and editing. Both authors have read and agree to the published version of the manuscript.
